# Throttling Growth Speed: Evaluation of *aux1-7* Root Growth Profile by Combining D-Root system and Root Penetration Assay

**DOI:** 10.3390/plants11050650

**Published:** 2022-02-27

**Authors:** Judith García-González, Jozef Lacek, Wolfram Weckwerth, Katarzyna Retzer

**Affiliations:** 1 Laboratory of Hormonal Regulations in Plants, Institute of Experimental Botany, Czech Academy of Sciences, 165 02 Prague, Czech Republic; garciago.judith@gmail.com (J.G.-G.); lacek@ueb.cas.cz (J.L.); 2 Department of Experimental Plant Biology, Faculty of Science, Charles University, 128 00 Prague, Czech Republic; 3 Department of Functional and Evolutionary Ecology, Molecular Systems Biology (MoSys), Faculty of Life Sciences, University of Vienna, Djerassiplatz 1, 1030 Wien, Austria; wolfram.weckwerth@univie.ac.at; 4 Vienna Metabolomics Center (VIME), University of Vienna, Djerassiplatz 1, 1030 Wien, Austria

**Keywords:** AUXIN-RESISTANT 1, AUX1, directional root growth, gravitropic response, mechanostimulus, mechanoadaptation, root skewing, root elongation rate, D-root system, root penetration assay

## Abstract

Directional root growth control is crucial for plant fitness. The degree of root growth deviation depends on several factors, whereby exogenous growth conditions have a profound impact. The perception of mechanical impedance by wild-type roots results in the modulation of root growth traits, and it is known that gravitropic stimulus influences distinct root movement patterns in concert with mechanoadaptation. Mutants with reduced shootward auxin transport are described as being numb towards mechanostimulus and gravistimulus, whereby different growth conditions on agar-supplemented medium have a profound effect on how much directional root growth and root movement patterns differ between wild types and mutants. To reduce the impact of unilateral mechanostimulus on roots grown along agar-supplemented medium, we compared the root movement of Col-0 and *auxin resistant 1-7* in a root penetration assay to test how both lines adjust the growth patterns of evenly mechanostimulated roots. We combined the assay with the D-root system to reduce light-induced growth deviation. Moreover, the impact of sucrose supplementation in the growth medium was investigated because exogenous sugar enhances root growth deviation in the vertical direction. Overall, we observed a more regular growth pattern for Col-0 but evaluated a higher level of skewing of *aux1-7* compared to the wild type than known from published data. Finally, the tracking of the growth rate of the gravistimulated roots revealed that Col-0 has a throttling elongation rate during the bending process, but *aux1-7* does not.

## 1. Introduction

Roots have evolved to grow in darkness and surrounded by soil along the gravity vector [1,2,3,4]. They adapt their growth direction and rate to their ever-changing environment, which includes changes in soil density or nutrient availability [5,6]. The root tip senses the pressure of a more compact soil and either adjusts the root thickness to penetrate it or changes the direction of growth [5,7]. Under drought conditions, the soil becomes more compact, and in addition to the limiting effects on root growth itself, mechanical impedance also restricts shoot growth, probably by increasing energy consumption [6]. Therefore, it is of agronomic importance to understand how these growth adaptations are modulated [8,9,10]. As recently described by Taylor et al. [6], root movement efficiency is fundamental to plant survival, but it is a complexly regulated collection of growth traits that are orchestrated by the interplay of multiple signaling pathways [6]. In addition to continuous growth along the gravitropic vector and the modulation of root system architecture to ensure efficient nutrient uptake, roots change their growth pattern form circumnutation to strict penetration depending on soil compaction [5,11,12,13]. This requires appropriate mechanostimulus perception, followed by signal transmission and mechanoadaptation [5,6,14,15]. The importance of orchestrated shootward auxin distribution along the root epidermis between the meristem and differentiation zone for the efficient modulation of root growth has been demonstrated repeatedly [3,4,16,17,18,19,20,21,22,23,24,25,26,27,28]. In 1990, Okada and Shimura [29] identified six *Arabidopsis thaliana* mutants with an apparent wavy phenotype, including the loss of function of a plasma membrane-located auxin influx carrier, AUXIN RESISTANT 1 (AUX1) [16,25,28,29,30,31,32,33,34]. The adaptation of root growth patterns is often studied by observing seedlings growing on an agar-enriched medium [35,36]. By increasing the percentage of agar in the growth medium and inclining the plates, the root tip experiences more pressure on the contact side between the root and the medium, which results in a wavy root growth pattern [7,26,35]. A loss of AUX1 also leads to root agravitropism and to a loss of perception of mechanical stress [7,25]. In addition, AUX1 is critical for the efficient circumnutation of rice roots through the soil [6]. AUX1 activity has been calculated as being able to enable the shootward auxin gradient, which is considered to orchestrate the spatial and temporal modulation of cell expansion in the elongation zone, to be established 10–20 times faster [16,24,25,28]. Because the root responds to mechanical stress by reducing its elongation rate and cell length, which likely allows for an increase in the root diameter to ensure better soil penetration, we speculated that AUX1 loss may negatively affect root velocity adaptation in response to mechanical impediment [37].

## 2. Results

### 2.1. Introducing the Combination of D-Rootsystem and Root Penetration Assay to Study Directional Root Growth Adaptation

**Figure 1:**
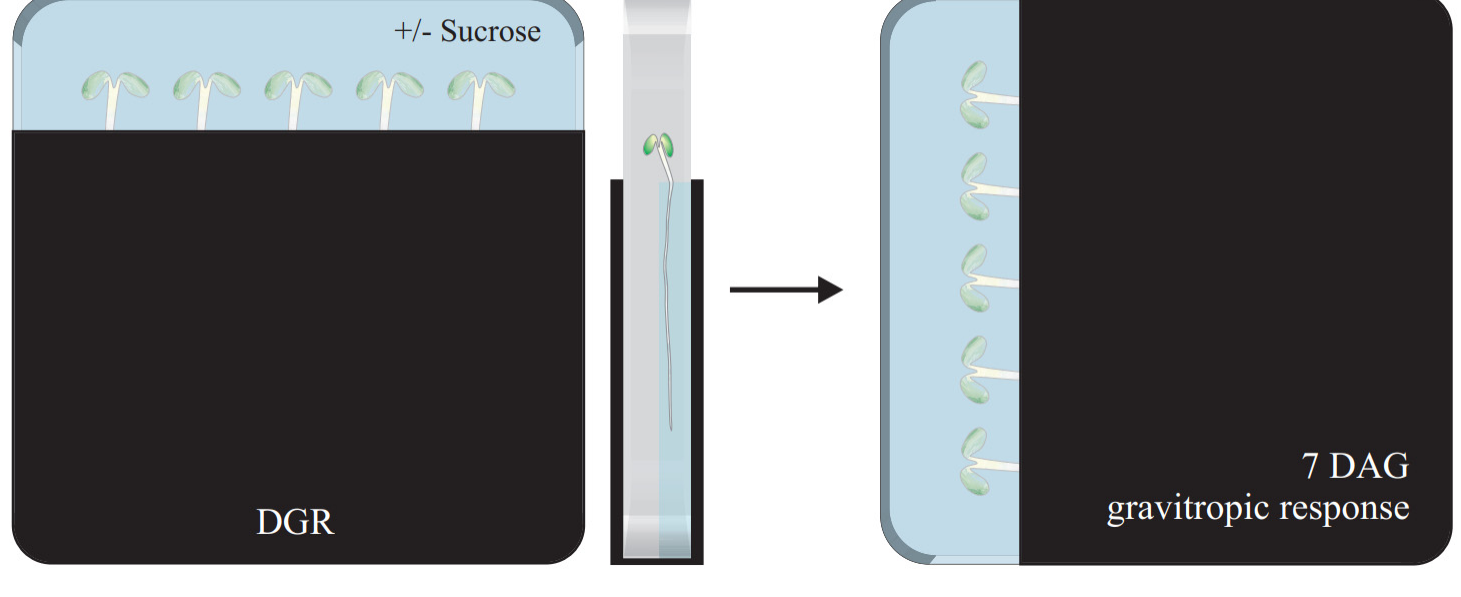
Schematic representation of the experimental setup. Briefly, seeds were placed on top of an agar layer unsupplemented or supplemented with sucrose. Plates were kept in the D–root system, allowing seedlings to grow with shaded roots for seven days. Primary root parameters (e.g., penetration frequency, root length, vertical growth index, straightness, and skewing angle) and gravitropic response were measured after a 90° turn. DGR: dark-grown root, DAG: days after germination.

Previously published studies have shown that wild-type roots reduce their growth rate when confronted with obstacles, whereas the *aux1* mutant shows no reduction in its root growth rate under mechanical stress conditions [7]. The experiments were performed on seedlings with their roots exposed to light during cultivation and growth along the medium’s surface. It is known that direct illumination, the stiffness of the agar supplemented medium, and the angle between the root tip and the presence of obstacles influence the modulation of directional root growth [4,7,26,35,38,39,40,41]. Therefore, we wondered how the *aux1* mutant would respond to uniformly applied mechanical stress compared to the wild type, and performed a so-called root penetration assay [42]. Furthermore, we complemented the root penetration assay with the D-root system, a device that allows to study roots that are shaded from direct root illumination (Figure 1). Recently, we published research that indicated that direct root illumination and sugar supplementation additively enhance the deviation of directional root growth, with sugar supplementation having a greater impact [39]. Direct root illumination triggers the so-called light escape mechanism, root elongation, but inhibits root meristem activity. Exogenous sucrose supplementation results in a more pronounced elongation and proliferation rate [43,44,45,46,47]. By stimulating the roots uniformly, reducing direct illumination, and testing the effect of sucrose supplementation, we aim to establish an experimental setup to analyze to what extent AUX1-mediated shootward auxin transport underpins the gravitropic response compared to mechanoadaptation.

### 2.2. Loss of AUX1 Results in Reduced Growth Medium Penetration Efficiency

**Figure 2:**
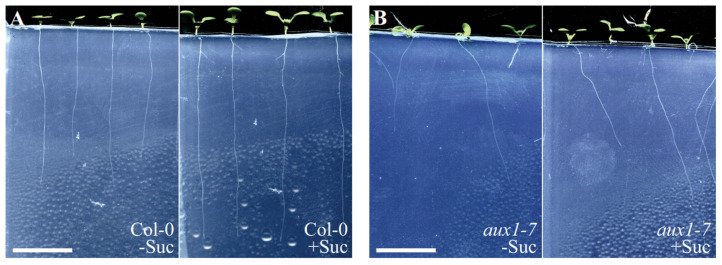
Representative images of seven DAG (**A**) Col-0 and (**B**) *aux1-7* seedlings grown in agar that had been unsupplemented or supplemented with sucrose. Scale bar = 10 mm.

Primarily, we compared the ability of the *aux1-7* and Col-0 roots to grow into ½ MS medium supplemented with 1% agar, with and without the addition of 1% sucrose. We removed a part of the medium to place the seeds on top of it and scanned the plates seven days after germination to evaluate the root penetration efficiency (Figure 2A,B).

**Figure 3:**
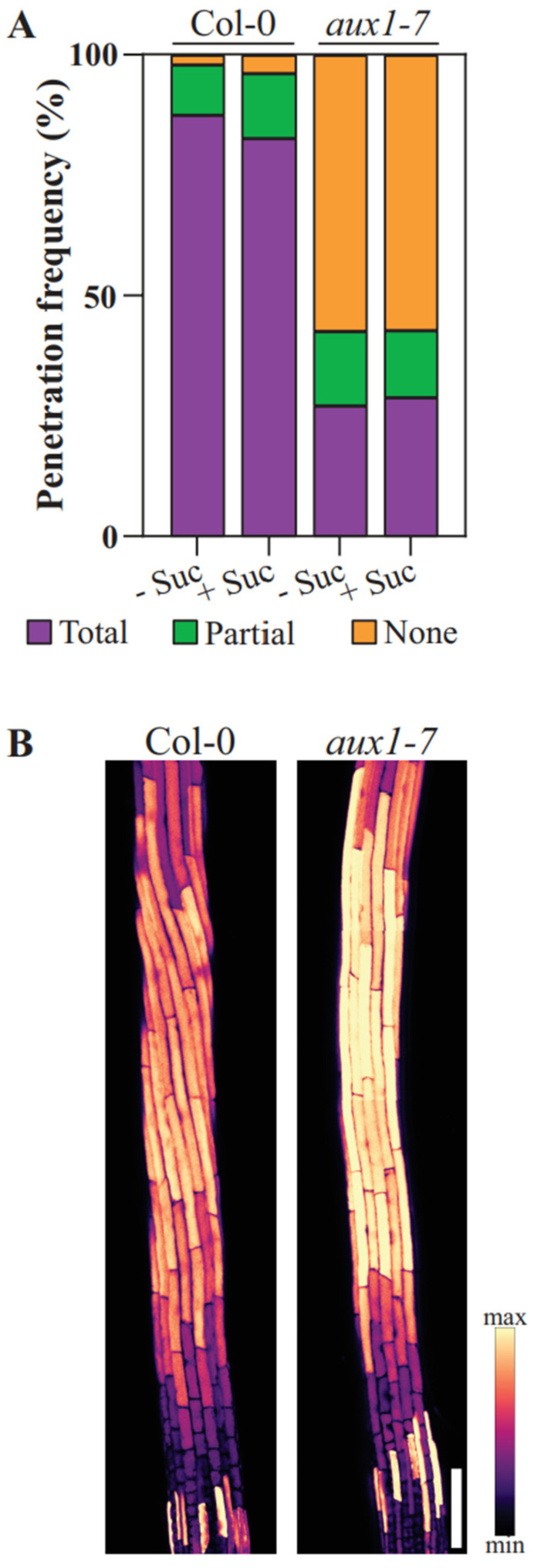
(**A**) Penetration frequency of seedlings grown in agar. Three categories were considered: no penetration (None), total penetration (Total; entire root embedded in the agar), and partial penetration (Partial; only part of the root can grow into the agar) (*n* = 42–57 roots). (**B**). Vacuole visualization in *Arabidopsis* roots with focus on the elongation zone. Staining with BCECF-AM was performed to distinguish individual root cells.

The addition of sucrose did not significantly change the root penetration frequency in either line (Figure 3A). However, compared to the wild type, only a fraction of the *aux1-7* roots (27.17% without and 28.89% with sucrose) succeeded in growing into the growth medium (Figure 3A). This suggests that the mutant struggles to grow into soil with increased compactness under the surface-exposed roots, consistent with the recently published study, which shows that AUX1 is critical for the efficient modulation of root movement [6]. Therefore, we examined the root morphology of the Col-0 roots that successfully penetrated the agar-supplemented medium and compared it to the *aux1-7* roots after staining them with the vacuolar stain BCECF-AM to visualize the individual cells. We found that every Col-0 root performs a twisting movement at the position of the root elongation zone, whereas *aux1-7* fails to organize its root shape at the elongation zone in the same manner (Figure 3B). We suppose that when *aux1-7* roots fail to orchestrate the spatial and temporal modulation of the elongation zone, it also has diminished ability to drill into the growth medium, which correlates with the low penetration efficiency compared to the Col-0 roots.

### 2.3. Loss of AUX1 Expectedly Results in an Uncoordinated Root Growth Pattern When Grown in Medium

**Figure 4:**
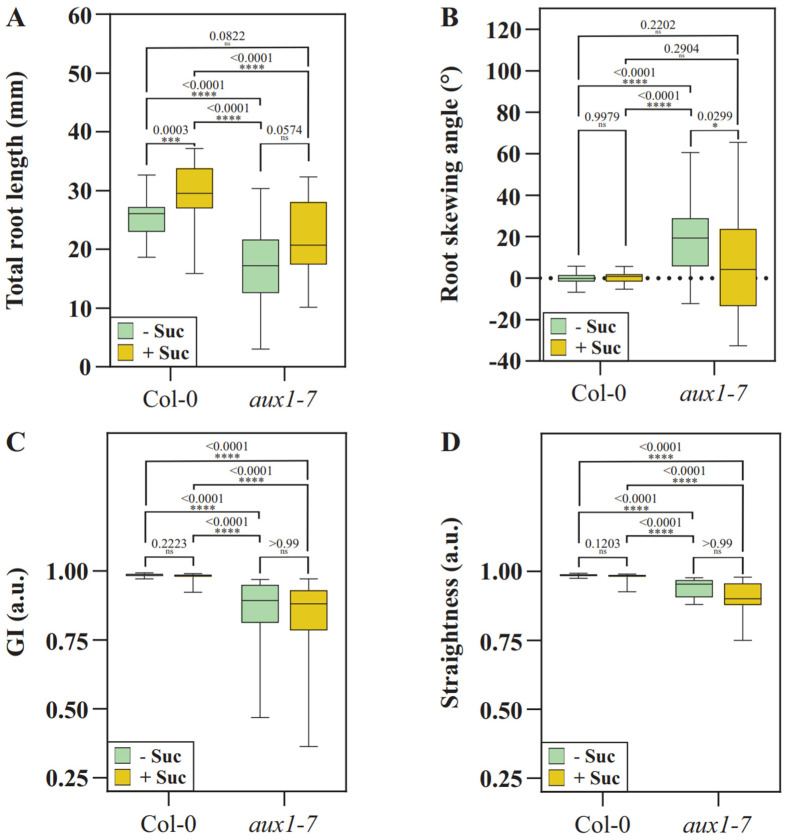
Analysis of root growth parameters: (**A**) root length, (**B**) skewing angle, (**C**) gravitropic index (GI), and (**D**) straightness. Only roots that showed total penetration into the medium were considered for analysis (Col-0, *n* = 38–50 roots; *aux1-7*, *n* = 15–16 roots). Statistical analysis: data normality was assessed through the Shapiro–Wilk test. Normally distributed data were analyzed with a One-Way ANOVA and Tukey’s post hoc multiple comparison test. Not normally distributed data were analyzed via a Kruskal–Wallis test followed by Dunn’s multiple comparison test. * *p* < 0.05; *** *p* < 0.001; **** *p* < 0.0001.

To test whether growth in medium enriched with 1% agar alters the already known growth differences between Col-0 and *aux1* mutants, we measured the total root length, root skewing angle, gravitropic index (GI), and straightness, which is also known as waviness. The total length of the primary root was used to reflect root growth efficiency [48]. When grown on medium, supplementing the medium with sucrose results in longer roots by increasing the cell proliferation rate [39,46]. Col-0 shows a significant increase in root length between the mediums with and without sucrose supplementation (Figure 4A). *aux1-7* grows shorter roots compared to Col-0, and sucrose supplementation does not result in a significant difference in root length, likely due to less auxin transport to the root meristem [16,28].

The root skewing angle reflects the slanted deviation of the root from the direction of gravity, something that *aux1* mutants have been previously described to do when grown exposed to light along a sugar-enriched growth medium [49,50]. In the current setup, Col-0 roots grow along the gravity vector in a less deviated manner compared to previously described experiments where the roots grew along the medium, and show no significant differences in the skewing angle and GI were found, regardless of supplementation (Figure 4B,C). Our previously published work showed that when roots are grown on the medium surface, there is a significant difference in the GI in response to the root illumination and sucrose supplementation status. We suspect that an uniformly perceived mechanical stimulus by wild-type roots that have grown into a medium limits the sugar-enhanced deviation from the vertical direction [27,39,46,51,52]. On the other hand, the *aux1-7* roots show a wider range of root skewing angles, while the difference between sucrose-enriched and non-enriched medium is small but still significantly relevant (Figure 4B). The deviation from the vertical direction is higher for *aux1-7* than it is for Col-0 in our penetration experiment (Figure 4C). We conclude that the directional growth of Col-0 deviates less because the roots grow in the dark and because the uniformly experienced mechanical stimulus limits the growth deviation, but *aux1-7* does not adapt to the mechanostimulus, which also correlates to the diminished ability to orchestrate the twisting movement at the position of the elongation zone (Figure 3A).

The wavy growth pattern, also referred to as straightness, reflects the ability of the root to respond to mechanic impedance [21,35,42,53]. Higher values for straightness indicate less curvature, fewer waves are formed, and lower values indicate more curvature [53]. Mutants with reduced shootward auxin transport were initially identified as roots lacking a wave pattern formation [26]. Published studies have described that Col-0 roots, when grown along the surface of a medium and with an increased plate inclination, exhibit a denser wave pattern that is further enhanced by the addition of sugar [26,35]. As in the case of GI, Col-0 roots show no significant difference in their waviness regardless of sucrose supplementation when embedded in the growth medium, and *aux1-7* roots show an expected uncoordinated growth pattern, resulting in a reduced straightness value (Figure 4D). Overall, the supplementation of ½ MS with 1% agar does not result in the growth medium having a high stiffness, meaning that root growth would be more impaired compared to the root growth along the medium, and the growth discrepancies between Col-0 and *aux1-7* described earlier are still present. We observed the largest differences in Col-0 when comparing the evaluated data with previously published differences in growth patterns that were associated with mechanosensing and adaptation depending on sugar supplementation [4,27,39,46,51,52].

### 2.4. Col-0 Roots Throttle Elongation Speed during Gravitropic Response, but Not aux1-7

**Figure 5:**
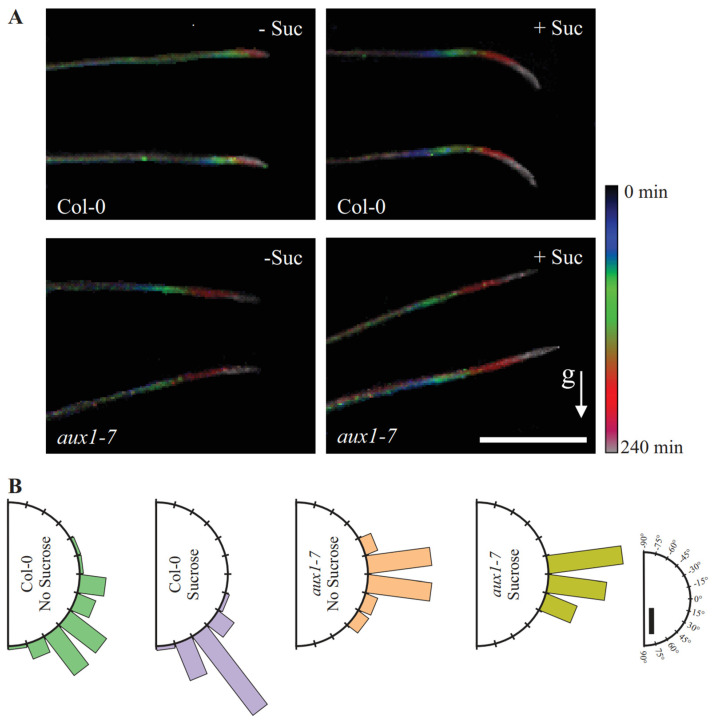
Gravitropic bending analysis. (**A**) Maximum projection of time-lapse imaging of root bending in unsupplemented and sucrose supplemented wild-type and *aux1-7* medium. Scalebar = 2 mm. Root imaging was carried out at time 0 (dark blue), 15, 30, 60, 120, 180, and 240 (light pink) minutes after turning the plates 90 degrees. (**B**) Root tip angle 240 min after gravistimulation. Roots were assigned to 15° sectors in a gravitropism diagram, and the percentage of roots belonging to each sector is represented by bars. Scalebar = 20%. (Col-0, *n* = 42–51 roots; *aux1-7*, *n* = 13–20 roots).

The loss of AUX1 results in agravitropic root growth and no gravitropic response, whereby the data were obtained from roots growing while exposed to light and along the surface of the medium’s surface [16,25,28,32,34]. We tested the gravitropic response of Col-0 and *aux1-7* roots in a combined D-root system and using a root penetration test approach. As expected and previously published, we tracked a pronounced bending curve over time for Col-0 and no response for *aux1-7* (Figure 5A).

**Figure 6:**
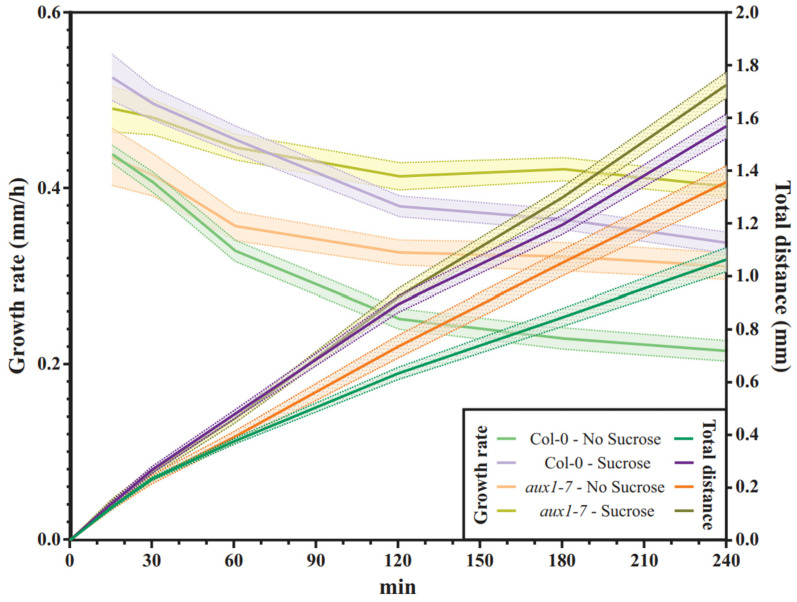
Root growth rates (left axis) compared to total growth distance (right axis) of 7DAG roots from the moment of gravistimulation over a period of four hours. Scans of the plates turned 90° were taken every 30 min, and the growth rate during the Col-0’s bending process compared to *aux1-7’s* was evaluated depending on sucrose supplementation (Col-0, *n* = 42–51 roots; *aux1-7*, *n* = 13–20 roots).

The maximum projection of the time-lapse images that we took every 30 min for four hours illustrates the form and growth rate of the bent root tips (Figure 5A). To quantify, if sucrose supplementation alters the bending efficiency of the roots when they grow into the agar, we analyzed the final root tip angle 240 min after gravistimulation, and it was determined that this was not the case (Figure 5B). However, regardless of the addition of sucrose, we observed that Col-0 limits the root growth rate during the bending process over time, while *aux1-7* roots continue to elongate at an almost constant rate (Figure 6). This is not surprising, as Fendrych et al. [24] have shown that cell elongation in roots is inhibited by exogenous auxin application and that this response requires the action of AUX1 [24,54]. This lack of the AUX1-dependent control of the root growth rate of individual cells in response to exogenously occurring signals may explain why *aux1* roots exhibit reduced root elongation control and diminished organization in the elongation zone (Figure 3B).

**Figure 7:**
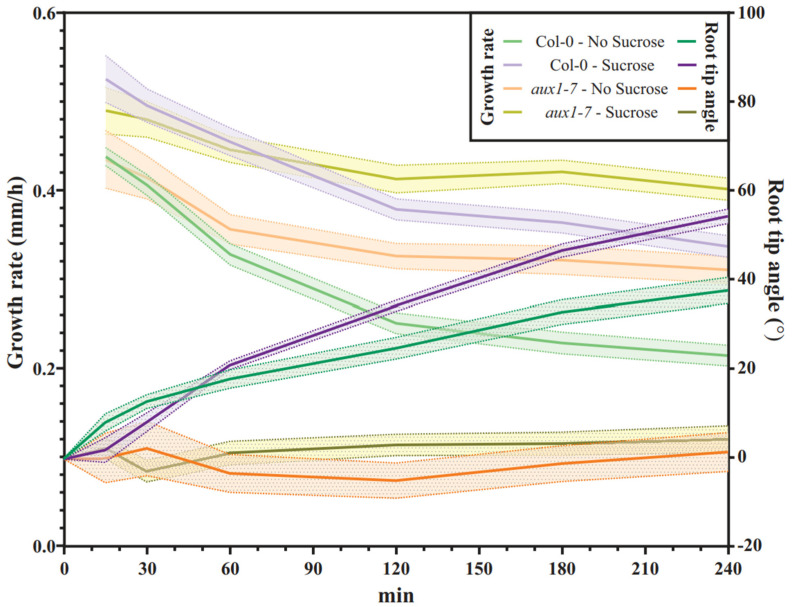
Time course of root bending upon gravistimulation of Col-0 and *aux1-7* 7DAG seedlings grown in agar with covered roots with or without sucrose supplementation. Root growth rates (left axis) versus root rip angles (right axis) are shown. (Col-0, *n* = 42–51 roots; *aux1-7*, *n* = 13–20 roots).

After losing the ability to modulate the elongation zone and the lack of throttling growth speed during the root bending process upon gravitropic stimulation, *aux1-7* roots do not respond to the gravitropic stimulus (Figure 7). Supplementing the growth medium with sucrose increases the growth distance over time (Figure 6), resulting in the bending angle of Col-0 grown in medium supplemented with sucrose is larger compared to that of roots grown on medium that had not been supplemented with sucrose (Figure 5A and Figure 7). Nevertheless, in both media, Col-0 is bends efficiently, whereas the *aux1-7* roots continue to grow in completely straight manner upon gravitropic stimulus (Figure 5B and Figure 7).

## 3. Discussion

Plant productivity and survival depend on efficient root growth in the soil. Roots have evolved to adapt and correct their architecture, volume, and directional root growth to ensure that enough water and nutrients are taken up to nourish the entire plant. For course direction, to avoid toxic compounds or obstacles, several signaling pathways are interwoven to initiate root growth adaptation.

Directional root growth adaptation is often studied by observing seedlings growing on agar-enriched medium [35,36]. By increasing the agar content and inclining the plates, the root tips experience more pressure and respond with a wavy root growth pattern [26]. Moreover, the addition of sucrose leads to increased deviation from vertical growth and from the straightness of the root when the roots grow along the medium [27,51,52]. The wavy pattern of the roots growing along the growth medium is orchestrated by multiple signaling pathways in the root tip, including mixed responses to gravisensing and mechanosensing [55]. Rutherford and Masson [56] proposed that thigmotropism is the cause of waviness and described that the changes in the symmetry at each half-wave occurs in response to gravity and mechanical impedance, with direction and force only varying for the mechanical stimulus, depending on the properties of the growth medium [56,57,58,59]. Gravity remains constant, and only the position of the plant can change relative to its source. Therefore, taller plants have evolved to grow their aboveground organs away (negative gravitropism) and their belowground organs along the gravitropic vector (positive gravitropism) [3,15,55,60,61,62].

With this study, we aimed to investigate the extent to which AUX1 activity maintains directional root growth under more natural growth conditions, namely omitting direct root illumination, sucrose supplementation, by combining the D-root system and the root penetration test. We chose the D-root system with and without sucrose supplementation because of the negative effects of direct root illumination and sugar supplementation shown in recently published studies [39,46]. Direct light illumination and exogenous sugar additively enhance the root growth that deviates from the vertical direction [39]. In addition, we chose to use the penetration test to uniformly expose the entire root to the same mechanostimulus intensity. In this way, we limited unnecessary exogenous stimuli by avoiding directional root illumination responding to uneven mechanostimuli and unnatural sucrose signaling in the case of plates without added sucrose.

Overall, we found a similar response in total root length growth and response to gravitropic stimulus as a function of the sucrose addition for Col-0 when the roots were grown in medium compared to published data for roots grown along surface of the medium. However, in contrast to the known data, we did not observe differential deviation from the vertical direction or increased waving of the Col-0 roots, which is generally observed in wild-type roots grown along a sucrose-enriched medium [27,47,52]. This is likely due to the ability of the wild-type roots to perform a twisting movement at the position of the elongation zone, which we could observe because the roots in our penetration assay are embedded in the medium and therefore are uniformly mechanostimulated but not impaired in the execution of their three-dimensional movement. The *aux1-7* roots do not show the same ability to modulate the elongation zone, which not only correlates with their reduced penetration ability and is further reflected in the loss of orchestrating directional root growth in general. Surprisingly *aux1-7* roots showed an unexpectedly high degree of skewness in the penetration assay, consistent with the recently suggested numbness of *aux1* mutants to mechanostimuli [7]. In addition, *aux1-7* root show a very low penetration frequency, with approximately 28% roots grown into the medium of all germinated seedlings, reflecting the importance of AUX1 as a mediator for efficient root movement [6]. Finally, when we tracked root growth during the bending assay, we found that *aux1-7* did not restrict the rate of root elongation compared to Col-0. Both lines responded as expected during the bending test and similarly to published results from roots grown on medium. The loss of root growth rate control in *aux1-7* is consistent with the observations of Fendrych et al. [24], who showed that AUX1 is required to limit root elongation upon exogenous auxin supplementation [24,63]. In summary, our results show that the combination of the D-root system and penetration assay using 1% agar-enriched ½ MS medium allows directional root growth to be observed while also reducing unnecessary interfering exogenous stimuli.

## 4. Materials and Methods

### 4.1. Plant Material and Growth Conditions

Col-0 and *aux1-7* seed stocks [32] were obtained from the Laboratory of Hormonal Regulations in Plants, Institute of Experimental Botany, Czech Academy of Sciences. Seeds were surface sterilized using 50% (*v*/*v*) bleach and 0.1% Tween20 (Sigma-Aldrich, Darmstadt, Germany) for 5 min and were then rinsed three times with sterile water. The seeds were plated on ½ Murashige and Skoog (Sigma) medium, solidified with 1% agar (Sigma), and adjusted to pH 6.0 by KOH. The medium was supplemented with either 1% sucrose (Merck-Millipore, Darmstadt, Germany) or was left without sugar [39]. To stimulate the roots to grow into the medium, its upper part at the border of the D-root system [38] was removed, and the seeds were placed on top [42]. The seeds were plated and stratified at 4 °C for two days before germination. The plates were positioned inclined at 45° from the vertical direction, at 22 °C and with a light intensity of 100 µmol/s/m^2^, in a climate-controlled growth room with long day conditions (16 h light, 8 h dark).

### 4.2. Root Parameter and Bending Analysis

Plate scans of seven-day-old *Arabidopsis thaliana* seedlings grown with covered roots were analyzed using the freely available ImageJ software. The root gravitropic index, straightness, and skewing angle were calculated according to Grabov et al. [53]. The skewing angle was determined based on the frontal orientation of the plates.

For the bending assay, 7DAG plants grown with covered roots were turned 90 degrees and were scanned at specific timepoints (0, 15, 30, 60, 90, 120, 180, 240 min). Images were aligned, and root tip coordinates were obtained using ImageJ. Data manipulation to determine the root tip angle and root growth rate was carried out using Microsoft Excel.

Statistical analysis and visual representation of the data were performed using GraphPad Prism. Normality was assessed via the Shapiro–Wilk test. Normally distributed data were evaluated with a One-Way ANOVA and Tukey’s post hoc multiple comparison test; not normally distributed data were analyzed with the non-parametric Kruskal–Wallis test followed by Dunn’s multiple comparison test.

Imaging was performed on five-day-old seedlings with roots grown in agar-supplemented medium without sugar supplementation, that had been shaded from direct light illumination, and that had been stained with BCECF-AM (10 μM, 60 min). The seedlings were placed on medium in a chambered coverslip, and pictures were taken with a Zeiss LSM880 laser scanning microscope in a horizontal setup that enabled the samples to be placed vertically using the objective 20×, at the Imaging Facility of the Institute of Experimental Botany AS CR.

## Data Availability

The data presented in this study are available in Supplemental Table S1.

## Supplementary Materials

The following supporting information can be downloaded at: https://www.mdpi.com/article/10.3390/plants11050650/s1, Table S1: raw data.
